# Preclinical evaluation of an mRNA-LNPs vaccine for mucosal protection against dental caries

**DOI:** 10.3389/fmicb.2026.1785919

**Published:** 2026-03-10

**Authors:** Yongchuan Zhou, Hong Shi, Qiong Guo, Yue Li, Lu Lv, Lili Hou, Cong Geng, Dazhuang Wang, Shaoxiong Yu, Shuai Ma, Yilin Li, Zhaopeng Sun, Chunlei Li

**Affiliations:** 1 School of Pharmacy, Hebei Medical University, Shijiazhuang, China; 2 CSPC Pharmaceutical Group Co., Ltd., Shijiazhuang, China; 3 Department of Pediatric Dentistry, Hebei Key Laboratory of Stomatology/Hebei Technology Innovation Center of Oral Health, School and Hospital of Stomatology, Hebei Medical University, Shijiazhuang, China; 4 Hebei Key Laboratory of Innovative Drug Research and Evaluation, School of Pharmaceutical Sciences, Hebei Medical University, Shijiazhuang, China; 5 Hebei Key Laboratory of Innovative Drug Research and Evaluation, Shijiazhuang, China; 6 State Key Laboratory of New Pharmaceutical Preparation and Excipients, Shijiazhuang, China

**Keywords:** dental caries, Fc fusion antigen, heterologous prime-boost, mRNA-LNPs vaccine, mucosal immunity, *Streptococcus mutans*

## Abstract

**Objective:**

Despite the success of mRNA-LNPs vaccines against viruses, their potential against prokaryotic pathogens remains underexplored. We provide proof-of-concept for an mRNA-LNPs vaccine against *Streptococcus mutans* (*S. mutans*), the primary cause of dental caries.

**Methods:**

We constructed LNPs-encapsulated mRNA vaccines encoding *S. mutans* PAc antigen alone or PAc fused with human IgG Fc domain, aiming to enhance mucosal immunity via Fc-FcRn interactions.

**Results:**

A heterologous intramuscular prime-intranasal boost regimen induced robust, durable (>4 months) sIgA responses-2.6-fold higher with Fc fusion-that significantly inhibited *S. mutans* biofilm formation *in vitro* and reduced moderate dentinal caries by >60% in rats.

**Conclusion:**

Bacterial antigens can be effectively delivered via mRNA platforms, and Fc fusion is a promising strategy to enhance mucosal immunity against oral, respiratory, and other mucosal pathogens, consistent with Fc-FcRn-mediated mechanisms.

## 1 Introduction

Dental caries remains a major global public health burden despite widespread use of fluoride, sealants, and oral hygiene programs. While these interventions reduce caries risk, they rely on consistent compliance and do not provide durable and active immune protection against *Streptococcus mutans* (*S. mutans*), the primary etiological agent (Patel, 2020). Many children, especially those in high-risk groups or low-access settings, continue to experience early childhood caries and recurrent lesions due to limited adherence or persistent bacterial colonization. A safe and effective mucosal vaccine that induces long-lasting salivary immunoglobulin A (sIgA) and mucosal immune memory could complement existing strategies by providing sustained protection, particularly for high-risk pediatric populations and individuals with repeated caries. This approach addresses a critical unmet need for interventions that reduce caries incidence and recurrence independently of daily compliance (Yan, 2013).

The mRNA-lipid nanoparticle (mRNA-LNPs) vaccines have achieved unprecedented success against viral pathogens, as exemplified by the rapid development and clinical efficacy of SARS-CoV-2 vaccines (Turner et al., 2021; Kim et al., 2022), yet applications targeting prokaryotic organisms remain scarcely reported. Vaccines for mucosal pathogens—whether bacterial, viral, or otherwise—requiring robust local immunity remain a major challenge. This is particularly evident for *S. mutans*, the primary etiologic agent of dental caries that affects billions globally (Kassebaum et al., 2015). Despite a century of effort since Bowen first proposed the concept of anti-caries immunization in 1969 (Batista et al., 2017), no effective vaccine has been successfully developed.

Protein antigen c (PAc) is a major virulence factor in dental caries and is associated with the early colonization of *S. mutans* on the surface of teeth (Okahashi et al., 1989). The screening of conserved sequences of antigenic proteins is the basis for the development of anti-caries vaccines (Cao et al., 2016). However, many analyses have shown that PAc conserved sequences have low immunogenicity and can induce the secretion only of unstable, transient and low-intensity secretory sIgA (Yan, 2013). To further enhance mucosal immunogenicity, we employed an Fc-fusion strategy. The Fc domain of human IgG binds to the neonatal Fc receptor (FcRn), which mediates pH-dependent transepithelial transport, improves antigen stability, and facilitates delivery to antigen-presenting cells (APCs) in mucosal tissues (Wieland and Ahmed, 2019). FcRn is abundantly expressed in nasal epithelium and APCs, enabling efficient transcytosis across mucosal barriers and enhanced antigen presentation (Guilliams et al., 2014). By fusing PAc to the Fc domain, we aimed to improve antigen persistence, mucosal uptake, and subsequent induction of protective sIgA and mucosal immune memory.

Despite decades of adjuvant and delivery system optimization, conventional anti-caries protein vaccines have failed to elicit potent, sustained salivary IgA (Patel, 2020; Yan, 2013). Parenteral mRNA vaccines alone similarly induce weak mucosal immunity, as systemic priming does not efficiently establish nasal-associated lymphoid tissue (NALT) memory (Mao et al., 2022). This highlights a critical unmet need for strategies that bridge systemic priming with mucosal recall to generate long-lasting immunity directly at mucosal surfaces (Ramirez et al., 2024; Kwon et al., 2025).

To address this, we engineered an mRNA encoding the *S. mutans* surface antigen PAc fused to the human IgG Fc domain. This novel design enhances mucosal delivery of mRNA-encoded antigens via FcRn-mediated uptake and immune activation in nasal epithelial cells and dendritic cells (Guilliams et al., 2014; Wieland and Ahmed, 2019; Roopenian and Akilesh, 2007). It represents a broadly applicable strategy for amplifying mucosal immunity against oral, respiratory, and other mucosal pathogens (Ochsner et al., 2021). By employing a heterologous intramuscular prime-intranasal boost regimen, we hypothesized that this platform would overcome the historical limitations of both protein and mRNA vaccines in mucosal bacterial infections (Mao et al., 2022; Sun et al., 2016).

In this study, we define mucosal immunity protection against dental caries as the induction of functional sIgA targeting *S. mutans*, reduced bacterial colonization on tooth surfaces and decreased caries lesion severity. We demonstrated that PAc-Fc mRNA-LNPs elicit durable (>4 months) sIgA, inhibit *S. mutans* biofilm formation, and provide substantial protection in a rat caries model. These findings establish the feasibility of mRNA vaccines for prokaryotic pathogens and validate Fc fusion as a translatable strategy to combat diseases requiring mucosal immunity.

## 2 Materials and methods

### 2.1 Ethics statement and animals

Female BALB/c mice (aged 6–8 weeks, body weight 18–22 g) and female Sprague–Dawley rats (aged 18 days, body weight 40–50 g) were obtained from Beijing Weitong Lihua Experimental Animal Technology Co., Ltd. (China) and housed under specific pathogen-free (SPF) conditions. All animal experimental protocols were subject to approval by the Animal Ethics Committee (LL-ZYZJ42407). The mice were kept in an environment with a 12-h light/dark cycle and subjected to a 12-h fasting period before administration. All animal experimental protocols were performed according to the ARRIVE Guidelines. Animals were randomly allocated to PBS, PAc mRNA-LNP, and PAc-Fc mRNA-LNP groups via a computer-generated random number table for balanced age and body weight; operators for mouse sample collection/ELISA (Section 2.5) and researchers for rat molar sectioning, staining, and Keyes caries scoring (Section 2.7) were blinded to group assignments to avoid subjective bias (Supplementary Table S1).

### 2.2 Construction of the fusion protein expression plasmid and mRNA synthesis

To target FcRn, we selected human IgG1 Fc. The rationale for using human IgG1 is that it has the highest affinity for activating FcγRI but the lowest affinity for inhibiting FcγRIIB (Nimmerjahn and Ravetch, 2005). Briefly, the gene sequence encoding the human IgG1 Fc domain (corresponding to the CH2-CH3 region of the IgG1 heavy chain, amino acid residues 231–447) (Elia et al., 2021) and the *pac* gene sequence encoding the amino acid residues 222 to 965 of the PAc protein from *S. mutans* UA159 (Guo et al., 2004) were amplified via PCR. A flexible (G4S)₃ linker (glycine-serine linker, sequence: GGGGSGGGGSGGGGS) was inserted between the PAc and Fc sequences to ensure proper folding and structural independence of the two domains. The fragments were subsequently cloned and inserted into the pVAX1 plasmid vector (Invitrogen, Catalog No.: V260-20, Lot No.: 230512) to construct the expression plasmids pVAX1-PAC and pVAX1-PAC-FC. All the expression plasmids were transformed into competent *E. coli* (JM108) (Beyotime, Catalog No.: C3012, Lot No.: 240125) and verified via DNA sequencing. *In vitro* transcription was performed using T7 RNA polymerase (Beyotime, Cat#M0251L, batch no.240109), with 1-methylpseudouridine (m^1^Ψ) replacing uridine to reduce innate immune activation of host cells. The synthesized mRNA contained a 5′ untranslated region (UTR) derived from human *α*-globin and a 3′ UTR derived from human α-globin, as well as a 120-nucleotide poly (A) tail to enhance mRNA stability and translation efficiency. The mRNA was capped using the CleanCap AG co-transcriptional capping method to ensure high translational activity. The final mRNA product was purified via high-performance liquid chromatography (HPLC) and stored at −80 °C until use.

### 2.3 Lipid-nanoparticle encapsulation of the mRNA

LNPs were formulated via microfluidic mixing (Geng et al., 2023). Briefly, an ethanol phase containing ionizable lipids (SM-102), DSPC, cholesterol, and DMG-PEG (50:10:38.5:1.5 mol ratio) was mixed with an aqueous phase (20 mM citrate buffer, pH 4.0) containing mRNA at a flow rate ratio of 1:3 (amine-to-phosphate ratio (N/P) of 6) in a microfluidic chip device. After mixing, LNPs were dialyzed against phosphate-buffered saline (PBS, pH 7.4) at 4 °C for 4 h using a dialysis membrane (molecular weight cutoff: 10 kDa) to remove ethanol and adjust the pH to physiological conditions. The particle size and particle distribution of the LNPs were assessed via Dynamic Light Scattering (DLS) on a Zetasizer Nano-ZS (Malvern, UK). The efficiency of mRNA encapsulation was determined through the Quant-iT RiboGreen RNA assay (Life Technologies, Cat#R11490, batch no.240108). The procedure entailed incubating the mRNA-LNPs at 37 °C for 10 min, both in the presence and absence of 1% Triton X-100 (Sigma-Aldrich, Cat#T8787, batch no.240102). Subsequent to the addition of the Ribogreen reagent, fluorescence intensities (Excitation/Emission: 480/520 nm) were recorded for the untreated samples (representing unencapsulated mRNA) and the samples treated with Triton X-100 (representing the total mRNA). The morphology of the LNPs was characterized via cryo-EM (Titan Krios, Thermo Fisher) with a K3 bioquantum (Gatan).

### 2.4 mRNA transfection

HEK293T cells were seeded in 6-well plates at a density of 6 × 10^5^cells per well and cultured in complete serum-containing medium (DMEM supplemented with 10% fetal bovine serum, 1% penicillin–streptomycin) in a humidified incubator. After 18 h of incubation (at ~70% confluency), the cells were transfected with 4 μg of mRNA-LNPs, and the transfection was maintained for 24 h under the aforementioned culture conditions prior to sample collection. For western blot analysis, total proteins were extracted using the ProteinExt® Mammalian Total Protein Extraction Kit (Beyotime, Cat#P0013, batch no.240116), followed by protein quantification with a BCA protein assay kit (Beyotime, Cat#P0009, batch no.240526). The extracted proteins were dissolved in 10% sodium dodecyl sulfate (SDS), and 25 μg of protein per lane was loaded for SDS-polyacrylamide gel electrophoresis (SDS-PAGE). After electrophoresis, the proteins separated in the SDS-polyacrylamide gel were transferred onto a nitrocellulose membrane Subsequently, the PVDF nitrocellulose membrane was blocked with PBS supplemented with 5% bovine serum albumin (BSA) for 2 h at room temperature, followed by washing with phosphate-buffered saline containing 0.1% Tween-20 (PBST) for three times. The membrane was then incubated with primary antibodies at 37 °C for 24 h. The anti-HA antibody (Abcam, Cat#ab9110, batch no.240135) and *β*-actin antibody (Abcam, Cat#ab8227, batch no.240136) were diluted at ratios of 1:1000 and 1:5000, respectively. After repeated extensive washing with PBST, the membrane was incubated with horseradish peroxidase (HRP)-conjugated secondary antibody (Abcam, Cat#ab236632, batch no. 240830) diluted at 1:2000 for 2 h, and then washed three times prior to three additional washes with PBS. For signal detection via enhanced chemiluminescence (ECL), The membranewas performed by incubating the membrane with either SuperSignal West Femto Maximum Sensitivity Substrate for 2 min for chemiluminescence development. Protein detection was carried out using the ImageQuant LAS4000 mini (Ge Healthcare Life Sciences), and the relative intensity of the protein bands was quantified using ImageJ software (National Institutes of Health, NIH).

### 2.5 Mouse immunization and PAc-specific antibody measurements

To study the immunization effect of PAc mRNA-LNPs and PAc-Fc mRNA-LNPs vaccines, female BALB/c mice (6–8 weeks, *n* = 6 per group) were immunized twice at an interval of 2 weeks. Seven immunization strategies involving 42 animals, namely, PBS, PAc vs. PAc-Fc IM + IM, PAc vs. PAc-Fc IN +IN, and PAc vs. PAc-Fc IM + IN, were included. The rationale for selecting the IM prime + IN boost regimen was to combine systemic immune priming (via IM administration, which induces strong systemic IgG responses) with mucosal immune enhancement (via IN administration, which targets nasal-associated lymphoid tissue (NALT) to promote local sIgA production). For IM administration, each mouse received 3 μg of mRNA encapsulated in LNPs in a total volume of 10 μL Tris buffer (pH 7.4), injected into the tibialis anterior muscle. For IN administration, each mouse received 10 μg of mRNA in 50 μL of buffer solution. 50 μL vaccine (10 μg PAc or PAc-Fc mRNA) was slowly dripped into both nostrils of the mice using a micropipette, and light inhalational anesthesia was induced with isoflurane to ensure adequate absorption of the solution. No acute inflammatory reaction was observed in the mice after immunization. Saliva and serum samples were obtained every 2 weeks after the initial immunization. Briefly, saliva was collected after stimulation of salivary flow by injecting each mouse via the intraperitoneal route with 20 μg of pilocarpine (Sigma-Aldrich, Cat#T0804-5MG, batch no.230403) in 0.1 mL of sterile PBS, with the saliva then being separated by centrifugation (14,000 rpm, 5 min). Blood samples were collected from retro-orbital plexus and clotted at room temperature, with the sera then being separated by centrifugation (4,500 rpm, 10 min).

PAc-specific sIgA and IgG antibody titers were calculated via ELISA (Yang et al., 2017). Briefly, Flat-bottom 96-well ELISA plates (Beyotime, Cat#P0301-12 × 8, batch no.240106) were coated with 100 μL of 3 μg/mL of rPAc 222–965 recombinant protein per well and incubated at 4 °C overnight. Plates were blocked with 1% bovine serum albumin in PBS for 2 h at 37 °C, and 100 μL of saliva (sIgA was detected by making 2-fold dilutions with PBST, and IgG was detected by making 5-fold dilutions with PBST) or serum (IgA was detected by making 5-fold dilution of PBST and IgG was detected by making 10-fold dilution of PBST) samples was subsequently applied in the blocking buffer. Alkaline phosphatase labeled goat anti-mouse IgG or IgA polyclonal antibodies (proteintech, Cat#SA00012-7, batch no.240330) and substrate p-Nitrophenyl phosphate (Sigma-Aldrich, Cat#T1503, batch no.240103) were used for detection. Absorbance was read by a microplate reader (Thermo Labsystems) at 405 nm. Positive criteria: Sample (OD_405_ nm) is greater than or equal to the average 2.1 of all blank control groups (the value of negative control sample is to take the reading value under the same dilution ratio as the sample to be tested).

### 2.6 Biofilm assay

Biofilm assays were carried out on 96-well microtiter plates as described previously with slight modifications (Pecharki et al., 2005). Briefly, overnight cultures of *S. mutans* were inoculated into a pre-warmed BHI medium and grew to an optical density at 600 nm of about 0.5 (approximately 1 × 10^8^ to 5 × 10^8^ CFU/mL). The cultures were diluted 1:100 in fresh BHI, and then 100 μL of the cell suspension with 100 μL of 20-fold BHI diluted serum or 5-fold BHI diluted saliva were incubated into the wells of a 96-well plate (Corning, USA). Plates were incubated at 37 °C in a 5% CO_2_ aerobic atmosphere for 16 h, during which the medium was not changed. For bacteria proliferation quantification, the media in the well were resuspended with a pipette and assessed by measuring the absorbance of suspension at 600 nm using a Microplate Spectrophotometer (Bio tek, USA). Media and unattached bacterial cells were gently poured out from the wells after lightly oscillating on a horizontal shaker, and then the plates were blotted on paper and air-dried. Adherent bacteria and derivatives were fixed with Bouin’s fixative for 2 h. After 2 times gently washing with PBS to remove Bouin’s fixative (Sigma-Aldrich, Cat#HT10132, batch no.240118), the biofilm on the bottom was stained with 100 μL of 0.1% crystal violet (Sigma-Aldrich, Cat#C3886, batch no.240110) for 15 min at room temperature. After two rinses with PBS, the bound dye was extracted from the stained cells by using 200 μL ethanol: acetone (4:1, v: v), and the plates were set on a horizontal shaker to allow full release of the dye. Biofilm formation was then quantified by measuring the absorbance of the solution at 570 nm. Bacterial colonization was assessed by measuring absorbance at 600 nm (OD₆₀₀) for planktonic growth and absorbance at 570 nm (OD₅₇₀) for biofilm biomass. All absorbance values were normalized to the Blank/PBS control group to account for baseline variation.

### 2.7 Anti-caries effect in rats

Three groups of female Sprague–Dawley (SD) rats were weaned at 18 days of age and fed with a cariogenic diet (Keyes, 1958). On the 20th day, chloramphenicol, ampicillin, and carbenicillin (1 g/kg of body weight) were added to the caries diet, and 200 mg/mL penicillin and 1,500 mg/mL streptomycin were added to the drinking water (antibiotic dosage was 1 g/kg) for 3 days. On the 23rd day, twelve rats were randomly divided into three groups (Blank, PBS, and PAc-Fc mRNA-LNP; n = 4 per group) using a computer-generated random number table. PBS and PAc-Fc mRNA-LNP groups of rats were inoculated with 2 × 10^9^ CFU of *S. mutans* via tooth surface smearing with a sterile cotton swab for 3 consecutive days (once a day). After confirming successful *S. mutans* infection in all challenged rats via oral swab culture and pac gene PCR verification, the PAc-Fc mRNA-LNPs group was immunized with 3 μg/10 μL PAc-Fc mRNA-LNPs via intramuscular injection, and on the 14th day after initial immunity, 80 μg/200 μL PAc-Fc mRNA-LNPs via nasal enhanced immunization, whereas the PBS group was immunized with PBS in the same way. On day 90, the mandibles were removed, cleaned, and stained with murexide (0.4% in 70% ethanol) (Sigma-Aldrich, Cat#M1253, batch no.240201). The molar teeth were washed and hemi-sectioned, and caries scoring was performed by a researcher blinded to group allocation (Keyes, 1958). Caries scoring criteria were defined as 0 (no caries) to 3 (moderate dental lesions). All caries scoring was performed by a single calibrated examiner blinded to group assignment.

### 2.8 Statistical analysis

All data analyses were performed using GraphPad Prism 9 software. Unpaired two-tailed *t* test was used to assess the statistical significance. A *p*-value of < 0.05 was considered as significant. Statistical significance was defined as *p < 0.05, **p < 0.01, ***p < 0.001 and ****p < 0.0001, with non-significant results denoted as n.s. (not significant).

## 3 Results

### 3.1 LNPs preparation, physicochemical characterization and characterization of PAc and PAc-Fc expression

**Figure 1:**
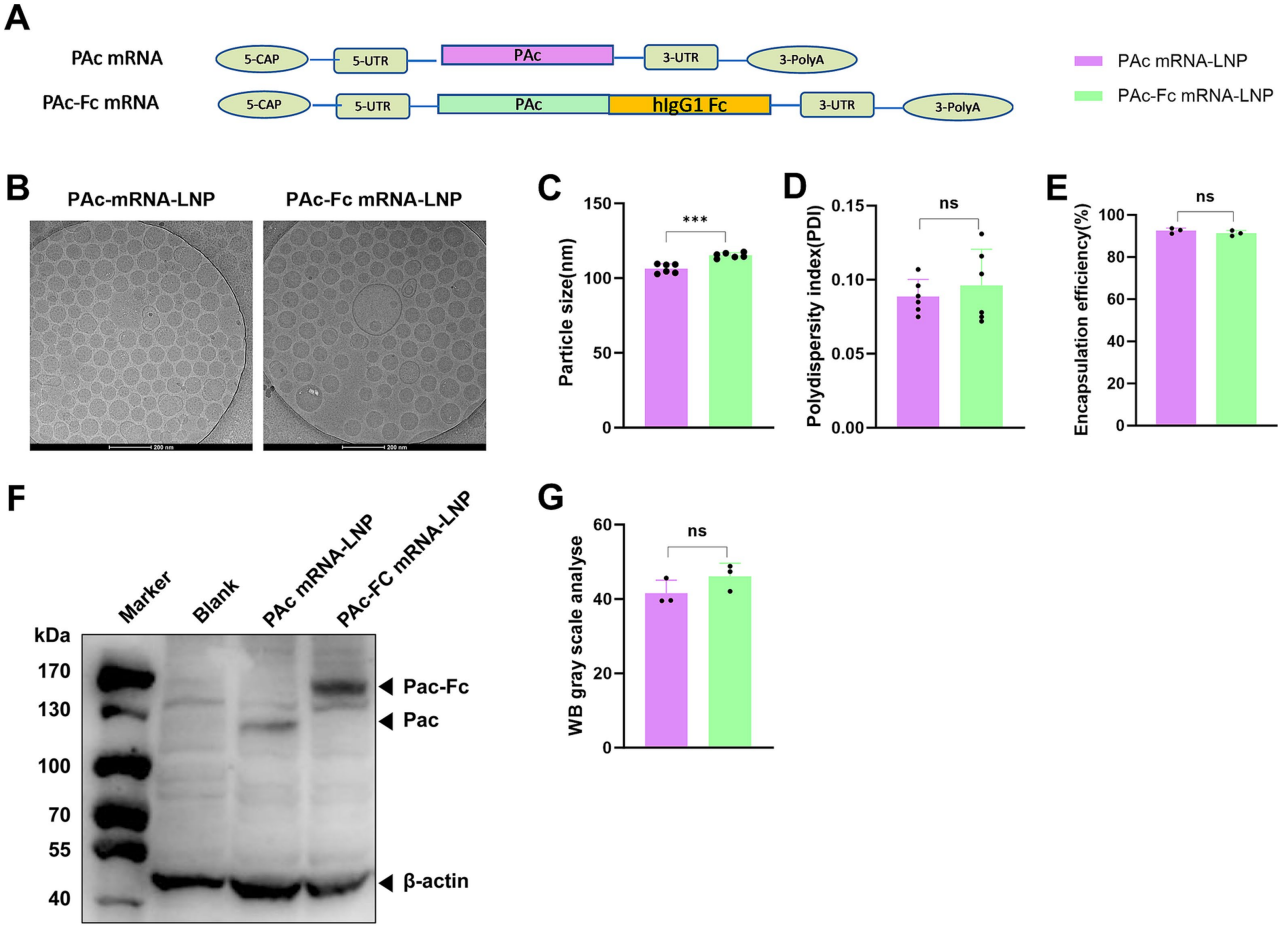
Design and physicochemical characterization of PAc-mRNA-LNPs\PAc-Fc mRNA-LNPs and subsequent expression characterization *in vitro*. **(A)** Design of the mRNA construct that expresses the antigens of PAc (222–965) and PAc-Fc. **(B)** A representative cryo-EM image of LNPs-encapsulated mRNA. Scale bar, 100 nm. **(C,D)** Particle size distribution and polydispersity index (PDI) of PAc and PAc-Fc mRNA-LNPs measured by dynamic light scattering (DLS). **(E)** mRNA encapsulation efficiency of PAc and PAc-Fc mRNA-LNPs determined by the Quant-iT RiboGreen RNA assay. **(F)** Western blot analysis of PAc and PAc-Fc protein expression in HEK293T cells transfected with corresponding mRNA-LNPs, probed with an anti-HA tag monoclonal antibody. **(G)** Grayscale quantitative analysis of Western blot bands. Three independent experiments were carried out in triplicates. Error bars represent standard deviations. All the data were analyzed using the Student’s *t* test; n.s., not significant, ***p < 0.001.

To study the mucosal immunity of mRNA-LNPs vaccines containing PAc and PAc-Fc antigens, we designed PAc and PAc-Fc mRNAs (Figure 1A). As shown in Figure 1B, cryogenic electron microscopy (cryo-EM) analysis revealed small and uniform particles. The LNPs containing PAc or PAc-Fc mRNAs had average effective diameters of approximately 100 nm or 110 nm, respectively (Figure 1C), a relatively uniform size profile, and polydispersity indices near 0.1 (Figure 1D). The encapsulation efficiency was similar for all the mRNAs tested, at approximately 90% (Figure 1E). The expression of the targeted antigen (PAc: ~80 kDa; PAc-Fc fusion protein: ~105 kDa) in HEK293T cells was then detected via western blotting with a hemagglutinin (HA) tag protein antibody. Western blot analysis revealed the expression of PAc and PAc-Fc proteins with the expected molecular size (Figure 1F; Supplementary Figure S1) and similar protein contents (Figure 1G), demonstrating that Fc fusion does not impair PAc antigen expression and both of the mRNA can be effectively translated into functional proteins. These results verify that the PAc and PAc-Fc mRNA-LNPs meet the requirement for subsequent immune evaluation.

### 3.2 The IM prime + IN boost is the optimal strategy for inducing persistent PAc-specific mucosal immunity

**Figure 2:**
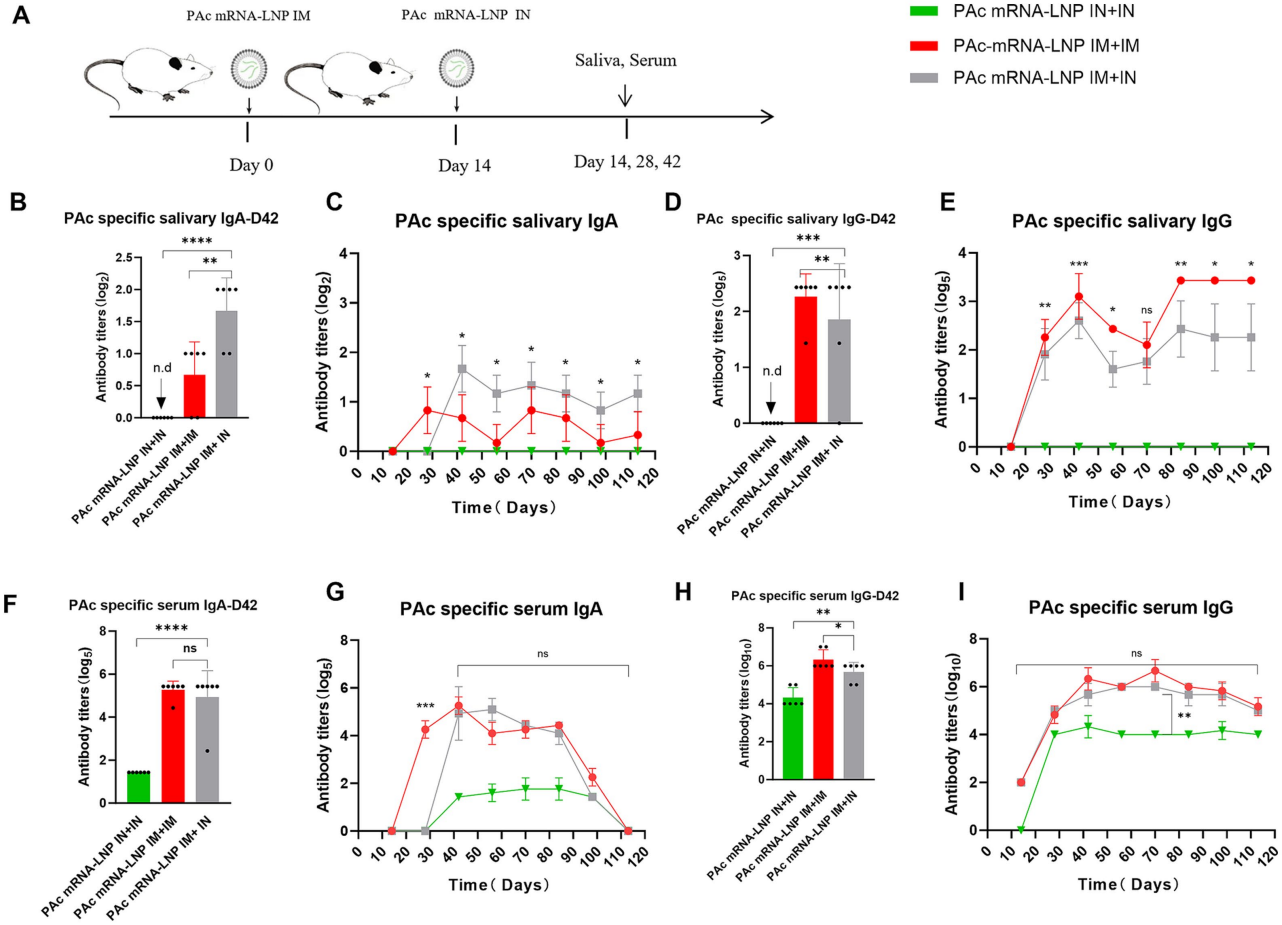
The PAc mRNA-LNPs vaccine induces PAc-specific mucosal immune responses via different immunization schedules. **(A)** BALB/c mice (*n* = 6) were intramuscularly immunized with 3 μg of mRNA-LNPs encoding PAc protein and intranasally immunized with 10 μg of PAc mRNA-LNPs. Serum and saliva samples were collected every 14 d after priming for 4 months to assess binding antibody responses. **(B–I)** Measurement of PAc-specific sIgA **(B,C)**, salivary IgG **(D,E)**, and serum IgA **(F,G)**, and serum IgG **(H,I)** in mice immunized with mRNA-LNPs IM + IM, mice immunized with mRNA-LNPs IN+IN, or mice immunized with mRNA-LNPs IM + IN. Statistical analysis was performed between Group PAc mRNA-LNPs IM + IN and Group PAc mRNA-LNPs IM + IM, with the results annotated in the time-course curves **(C,E,G,I)**. Three independent experiments were carried out in triplicates. Error bars represent standard deviations. All the data were analyzed using the Student’s *t* test; n.d., not detected, n.s., not significant, *p < 0.05, **p < 0.01, ***p < 0.001.

To identify the optimal immunization regimen for maximizing mucosal immune responses (a prerequisite for evaluating the effect of Fc fusion), we compared the impact of different immunization routes (IM and IN) and prime-boost combinations on PAc-specific mucosal immunity. We collected saliva and serum every 2 weeks after primary immunization and determined PAc-specific IgA and IgG antibody levels (Figure 2A). These levels followed a hierarchical pattern with PAc-specific sIgA levels depending on the immunization route in the following order: IM + IN > IM + IM > IN+IN. Saliva sIgA antibody titers induced by IM + IN immunization were twice as high as those induced by the IM + IM strategy and were equally persistent (Figures 2B,C). Mucosal immunity could not be induced by intranasal administration of PAc mRNA-LNPs alone (Figures 2B–E). However, IM + IM induced the highest salivary IgG titers (Figures 2D,E), indicating the important role of IM injection in the mRNA-LNPs vaccine. In addition, there was no significant difference in the serum IgA (Figures 2F,G) or IgG (Figures 2H,I) levels between the IM + IM group and the IM + IN group, indicating that the serum antibody levels were related mainly to the initial immunity of the IM. Moreover, the levels of serum IgA (Figures 2F,G) and IgG (Figures 2H,I) in the IN+IN group were also low, further confirming the advantage of IM priming in inducing systemic immunity.

We further analyzed the antigen-specific IgG1 (Th2 response) and IgG2a (Th1 response) levels in the serum (Supplementary Figure S2A). The levels of serum IgG1 and IgG2a antibodies in the IN + IN group were low, but the group had the highest Th2-linked subtype profiles. Compared with the IN+IN group, both the IM + IN and IM + IM groups presented higher levels of IgG1 and IgG2a antibodies, and the IM + IN group presented a greater titer of IgG2a antibodies and the most balanced Th1/Th2 response (Supplementary Figures S2B–D), suggesting that IM + IN not only induces strong mucosal immunity but also a balanced systemic immune response.

To further improve mucosal immunity, 1, 10, and 20 μg doses of PAc mRNA-LNPs were used for nasal administration for secondary immunization, and the results revealed that both the 10 μg and 20 μg groups presented significant improvements in mucosal immunity, with a stronger effect observed in the 20 μg group (Supplementary Figure S3A). However, during the experiment, there was obvious weight loss among the mice that received the 20 μg dose (Supplementary Figure S3C), indicating the potential toxicity. Therefore, we carried out the remaining experiments with a 10 μg dose to ensure safety while maintaining effective mucosal immunity induction.

### 3.3 Fc fusion antigen further enhances the sIgA response in the IM + IN immunization strategy

**Figure 3:**
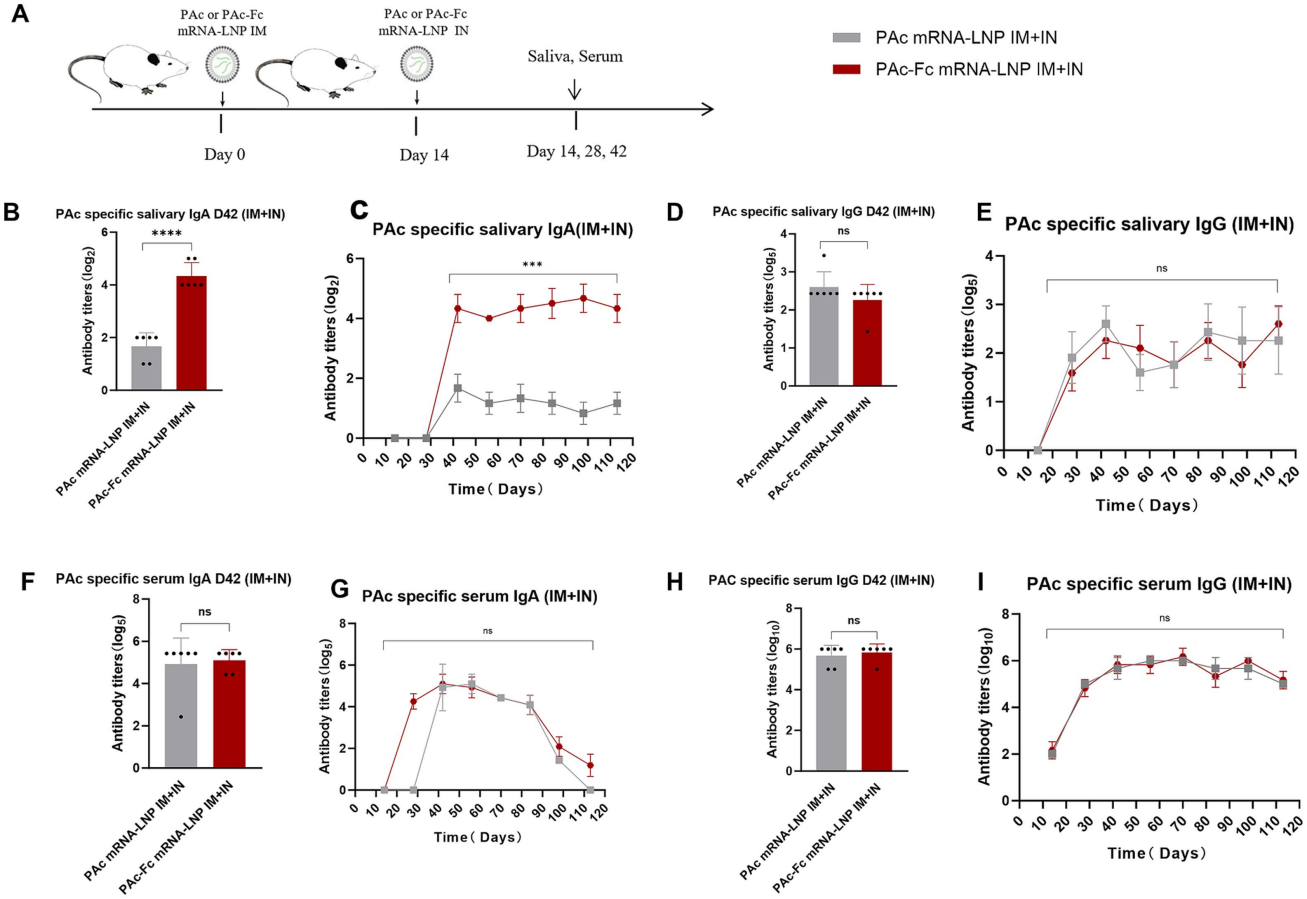
PAc-Fc mRNA-LNPs vaccination enhances PAc-specific sIgA in the IM (prime) + IN (boost) schedule. **(A)** BALB/c mice (*n* = 6) were vaccinated on an IM (prime) + IN (boost) schedule (d 0, 14). The mice were IM primed with 3 μg of PAc mRNA-LNPs, PAc-Fc mRNA-LNPs or IN boosted with 10 μg of PAc mRNA-LNPs or PAc-Fc mRNA-LNPs. Serum and saliva samples were collected every 14 d after IM priming for 4 months to assess binding antibody responses. **(B–I)** PAc-specific sIgA **(B,C)**, salivary IgG **(D,E)**, serum IgA **(F,G)**, and serum IgG **(H,I)** levels were measured in mice immunized with the mRNA-LNPs or PAc-Fc mRNA-LNPs IM + IN schedule. Three independent experiments were carried out in triplicates. Error bars represent standard deviations. All the data were analyzed using the Student’s *t* test; n.s., not significant, ***p < 0.001.

To test our core hypothesis that Fc fusion enhances PAc-specific mucosal immunity, especially salivary sIgA responses, under the optimal IM + IN regimen without adverse effects on systemic immunity, we directly compared PAc-Fc mRNA-LNPs (experimental groups), PAc mRNA-LNPs (experimental groups), and PBS (blank control) (Figure 3A). The results revealed significantly higher titers of saliva sIgA in the PAc-Fc mRNA-LNPs IM + IN group than in the PAc mRNA-LNPs IM + IN group (Figure 3B), directly confirming that Fc fusion enhances mucosal sIgA responses. Long-term observation revealed that the antibody titers induced by the vaccine were equally durable in both groups (>4 months) (Figure 3C). The results showed that PAc-Fc mRNA-LNPs induced the highest titer and long-lasting sIgA antibody response under IM + IN administration. Compared with the PAc mRNA-LNPs vaccine, the administration of the PAc-Fc mRNA-LNPs vaccine did not significantly influence the titers of saliva IgG, serum IgA, or serum IgG (Figures 3D–I), indicating that Fc fusion specifically enhances mucosal sIgA responses without altering systemic antibody levels or non-target mucosal IgG responses. However, compared with the PAc mRNA-LNPs vaccine, the PAc-Fc mRNA-LNPs vaccine induced a lower titer of IgG1, the same titer of IgG2a antibodies, and a more balanced Th1/Th2 response (Supplementary Figures S2B–D), suggesting that Fc fusion may fine-tune the systemic immune response.

In addition, we tested the immune effects of the Fc-fused antigen on IM and IN administration (Supplementary Figure S4A). Two-dose IM immunization with PAc-Fc mRNA-LNPs did not induce significantly higher titers of saliva sIgA than did two-dose IM immunization with PAc mRNA-LNPs (Supplementary Figures S4B,C). Conversely, a robust and statistically significant elevation in the sIgA titer was observed when two-dose IN immunization with PAc-Fc mRNA-LNPs compared with PAc mRNA-LNPs (Supplementary Figures S4D,E), indicating that Fc fusion enhances mucosal immunity preferentially under mucosal (IN) immunization conditions.

Finally, similar to the PAc mRNA-LNPs vaccine, increasing the intranasal dose of PAc-Fc mRNA-LNPs from 10 μg to 20 μg resulted in a statistically significant difference (mean ± SE: 10 μg = 4.33 ± 0.47; 20 μg = 4.5 ± 0.5; two-way ANOVA with Bonferroni correction, *p* = 0.038 < 0.05) but a modest magnitude of increase in mucosal immune response (Supplementary Figure S3B), indicating that higher intranasal doses do not substantially enhance the mucosal immunity. Moreover, during the experiment, there was obvious weight loss among the mice that received the 20 μg dose (Supplementary Figure S3C), indicating potential toxicity. Therefore, we carried out the remaining experiments with a 10 μg dose to ensure safety while maintaining effective mucosal immunity induction. Histopathological examination of heart, liver, spleen, lung, and kidney tissues with hematoxylin–eosin (HE) staining showed no obvious histological toxicities (e.g., inflammation, necrosis, congestion, or structural damage) in any dose group compared with the blank control group (Supplementary Figure S3D).

### 3.4 Vaccine-induced antibodies from PAc-Fc mRNA-LNPs immunization potently inhibit *Streptococcus mutans* biofilm formation *in vitro*

**Figure 4:**
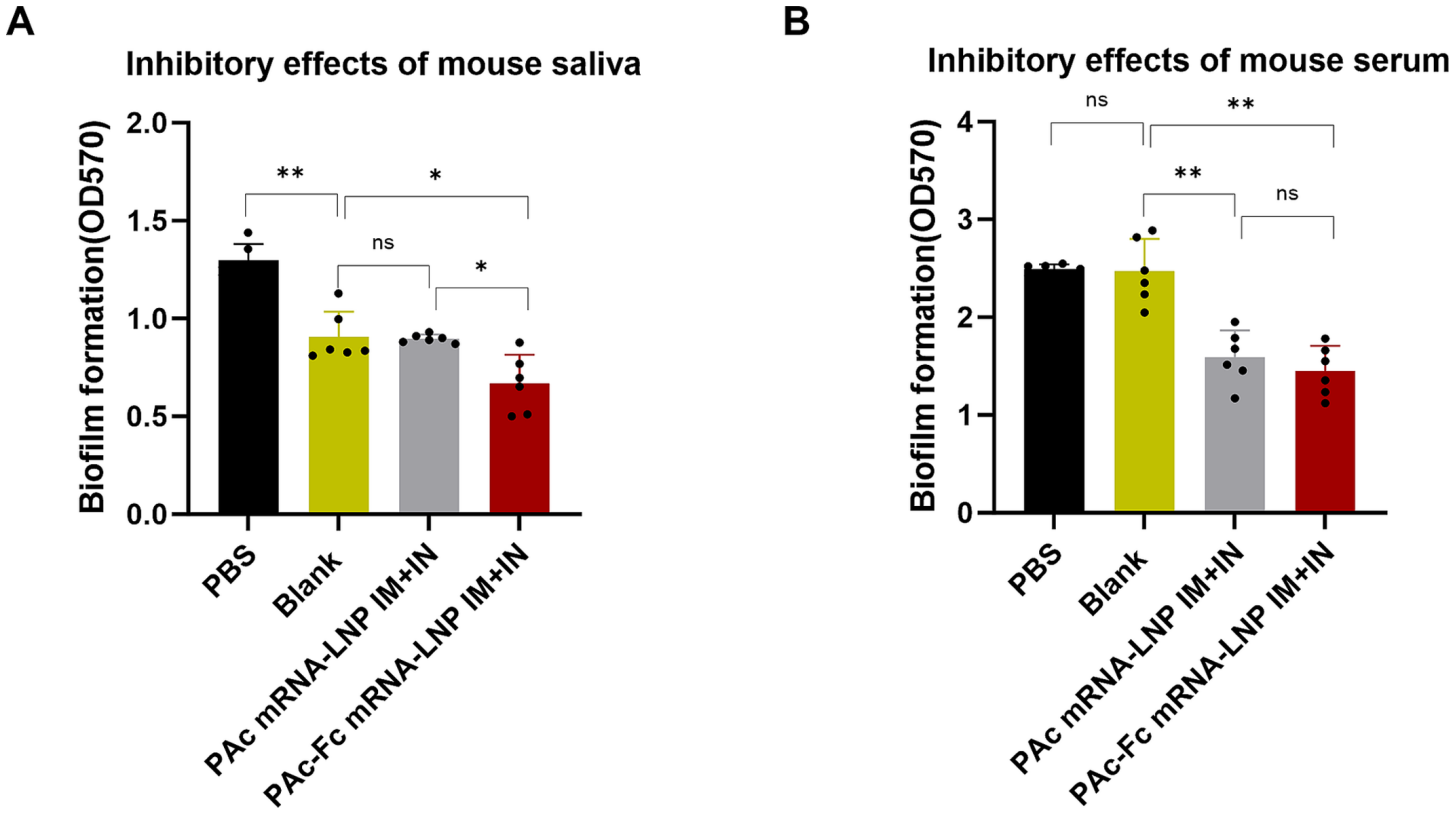
Inhibitory effects of mouse serum or saliva on *S. mutans* biofilm formation. In total, 100 μL of BHI-diluted mouse serum or saliva was mixed with 100 μL of BHI-diluted *S. mutans* and incubated for 16 h. Biofilm formation was quantified by measuring the extracted crystal violet stained to plate adherent bacteria and derivatives at 570 nm. Inhibitory effects of PBS, blank (saliva or serum from a mouse in the blank group), 5-fold diluted saliva (PAc mRNA-LNPs, sIgA titer = 2^1.5^; PAc-Fc mRNA-LNPs, sIgA titer = 2^4^) **(A)** and 20-fold diluted mouse serum (PAc mRNA-LNPs, IgA titer = 10^4^; PAc-Fc mRNA-LNPs, IgA titer = 10^4^) **(B)** from immunized mice. Three independent experiments were carried out in triplicates. Error bars represent standard deviations. All the data were analyzed using the Student’s *t* test; n.s., not significant, *p < 0.05, **p < 0.01.

To link enhanced mucosal immunity to functional anti-bacterial activity, we tested whether the elevated salivary sIgA responses induced by PAc-Fc mRNA-LNPs (IM + IN regimen) confer stronger inhibition of *S. mutans* biofilm formation compared with PAc mRNA-LNPs and blank controls. We evaluated this by directly comparing saliva and serum from PAc-Fc mRNA-LNPs, PAc mRNA-LNPs, and blank control groups. We tested the effects of PAc-specific immune responses to the mRNA-LNPs vaccine on protection against *S. mutans* infection. Saliva obtained from the PAc-Fc mRNA-LNPs IM + IN group strongly inhibited the biofilm formation of *S. mutans* compared with saliva obtained from the blank control group and PAc mRNA-LNPs IM + IN group (Figure 4A), which correlates with the higher salivary sIgA titers in the PAc-Fc group and confirms that Fc-enhanced mucosal immunity translates to improved anti-biofilm activity. The sera of the PAc-Fc mRNA-LNPs IM + IN group and PAc mRNA-LNPs IM + IN group, with similar antibody titers of IgA and IgG, also showed similarly significant effects effects on the inhibition of biofilm formation compared with those of the blank serum controls (Figure 4B), indicating that systemic antibodies contribute to anti-biofilm activity but Fc fusion does not further enhance this effect (consistent with the similar systemic antibody levels between the two groups).

### 3.5 Protection against caries formation

**Figure 5:**
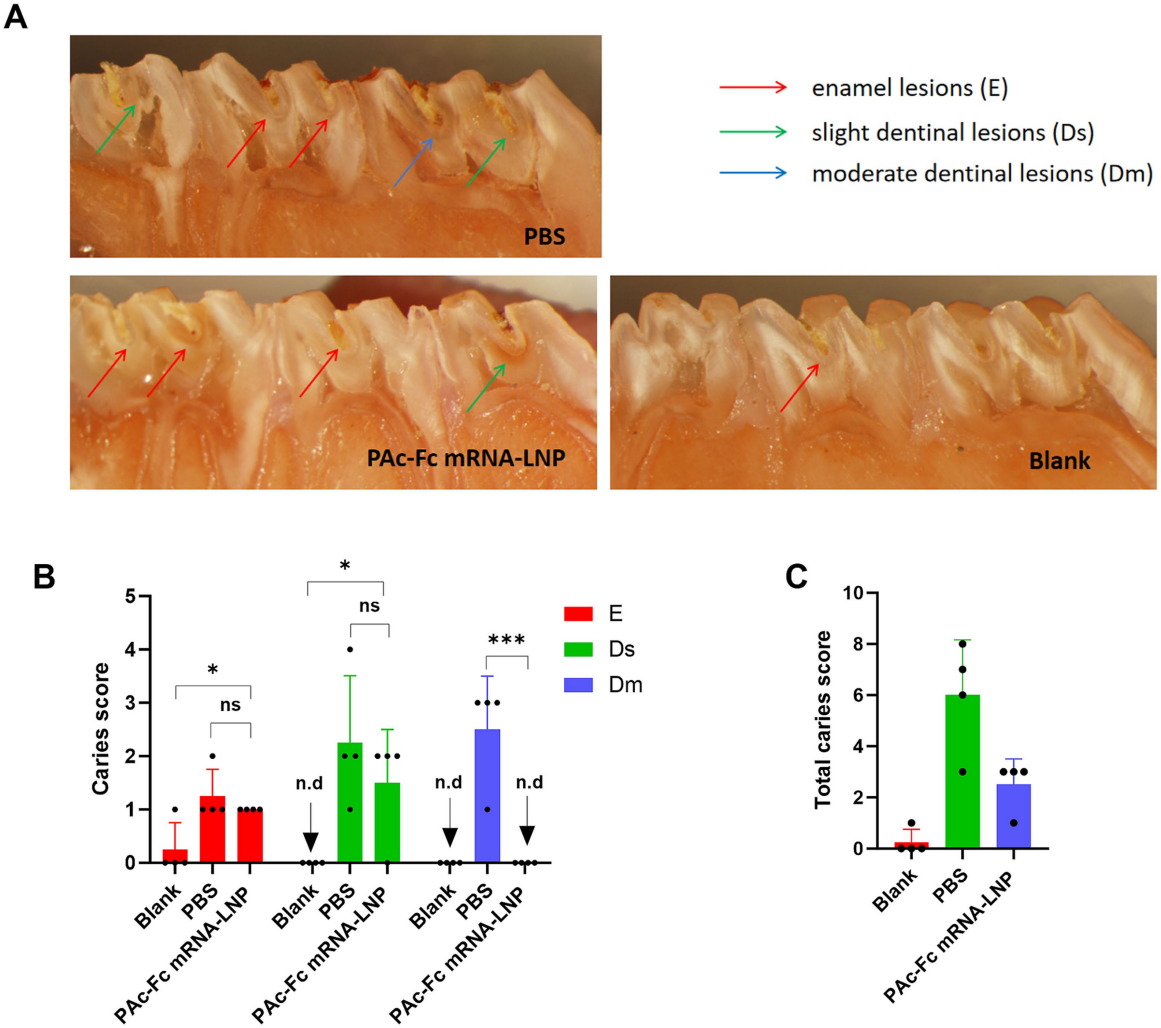
Inhibitory effects of PAc-Fc mRNA-LNPs on caries lesions. **(A)** Representative micrographs of caries lesions on molars from rats in each group. Enamel lesions (E), slight dentinal lesions (Ds), and moderate dental lesions (Dm) is indicated by red, green, or blue arrows, respectively. **(B)** Caries scores of E, Ds, and Dm for rats immunized with different group. **(C)** Total caries scores of different groups. Three independent experiments were carried out in triplicates. Error bars represent standard deviations. All the data were analyzed using the Student’s *t* test; n.d., not detected; n.s., not significant; *p < 0.05; **p < 0.01; ***p < 0.001.

To verify the *in vivo* protective effect of Fc-enhanced mucosal immunity—mediated by elevated salivary sIgA induced by the PAc-Fc mRNA-LNPs vaccine—we tested whether PAc-Fc mRNA-LNPs (IM + IN regimen) provides better protection against *S. mutans*-induced dental caries compared with PBS controls. We designed another separate experiment to evaluate the experimental dental caries which consisted of the three groups described earlier. The extension and depth of carious lesions were scored as enamel (E), slight dentinal (Ds), and moderate dental (Dm) involvement. PAc-Fc mRNA-LNPs immunized rats were protected against caries with fewer caries lesions compared with PBS group (Figure 5A), directly supporting that Fc-enhanced mucosal immunity confers in vivo protective effects. Furthermore, rats immunized with PAc-Fc mRNA-LNPs group showed significantly fewer Dm caries lesions than the PBS group. No significant difference in E or Ds caries lesions were observed in rats immunized with PAc-Fc mRNA-LNPs and PBS (Figure 5B). Accordingly, as shown in Figure 5C, significantly lower total caries scores (E + Ds + Dm) were recorded for the rats immunized with PAc-Fc mRNA-LNPs than for those immunized with PBS, confirming the overall protective effect of PAc-Fc mRNA-LNPs against dental caries.

## 4 Discussion

**Figure 6:**
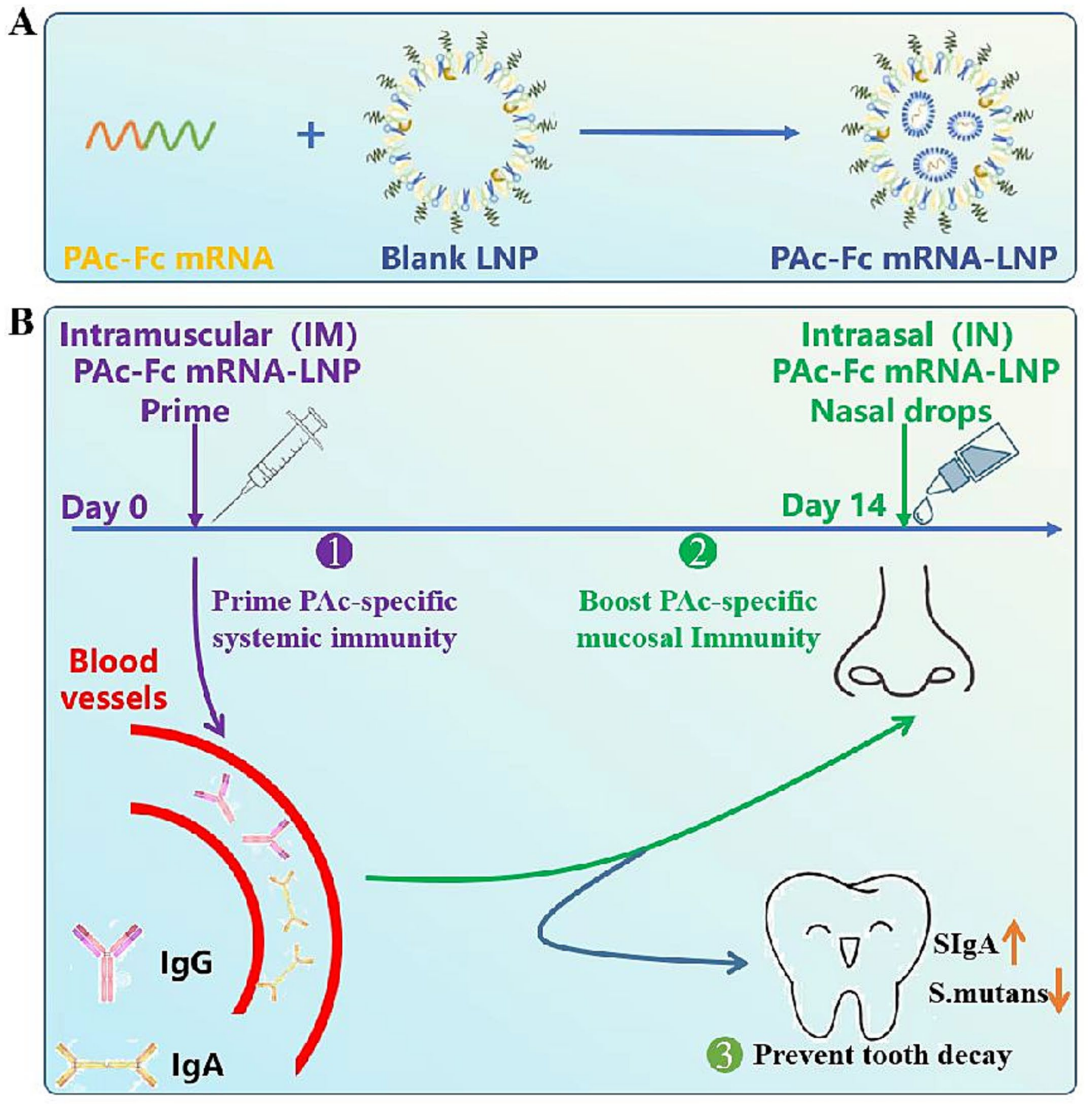
PAc-Fc mRNA-LNPs vaccine with Fc molecule fusion and its proposed mechanism of action (consistent with FcRn biology) to elicit potent mucosal immunity. **(A)** PAc-Fc mRNA-LNPs vaccine is formulated with lipidoid, phospholipid, DMG-PEG and cholesterol, along with PAc-Fc mRNA encoding the PAc-Fc fusion protein. **(B)** mRNA-LNPs vaccine encoding a PAc-Fc fusion antigen, delivered via a combined intramuscular (IM) prime and intranasal (IN) boost regimen, effectively induces a potent and durable sIgA response in mice.

Since Bowen first proposed the concept of immune anti-caries in 1969, various anti-caries vaccines have been developed, including protein vaccines, polypeptide vaccines, and DNA vaccines (Batista et al., 2017; Childers et al., 2006; Shi et al., 2012). However, anti-caries antigen proteins exhibit inherent low immunogenicity, consistently inducing variable, transient, and low-titer sIgA responses. Even with optimization of carriers, adjuvants, and administration routes, mucosal immune efficacy remains suboptimal (Yan, 2013). The novel coronavirus mRNA-LNPs vaccine SYS6006, which included ionizable lipids, DSPC, cholesterol, and mPEG-DMG-2 K (50:10:38.5:1.5 mol ratio), was developed by our research group and has been successfully developed in China. Intramuscular administration of SYS6006 was found to reduce the incidence of novel coronavirus pneumonia successfully but could not effectively induce mucosal immunity (Xu et al., 2023). In this study, we have developed, for the first time, an mRNA-LNPs vaccine capable of inducing long-lasting and highly effective mucosal immunity for preventing dental caries in a rat model (Figures 6A,B).

Vaccines administered via the mucosa can induce antigen-specific antibodies in the mucosal area, in which sIgA plays a major protective role in the upper respiratory tract and prevents microbial infection. Except for inhibiting bacterial adhesion and neutralizing viruses (both extracellularly and within epithelial cells) and bacterial toxins, the resistance of sIgA to proteases makes these antibodies uniquely suited for functioning in mucosal secretions (van Ginkel et al., 2000; Tang et al., 2022). The mechanism of action of salivary sIgA antibody against *S. mutans* is to interfere with its sugar-independent and sugar-dependent attachment and adhesion on tooth surfaces. Notable progress has been made in inducing sIgA antibodies targeting *S. mutans* surface adhesion proteins. For example, Yan (2013) developed a Flagellin-rPAc anti-caries vaccine and demonstrated that saliva and serum from immunized mice inhibit *S. mutans* biofilm formation *in vitro* (Sun et al., 2016). Consistent with these findings, our study shows that *S. mutans* biofilm formation was significantly inhibited when sIgA titers reached 16-fold above baseline, potentially attributable to synergistic effects with concurrent elevated salivary IgG titers. The study of nasal administration of recombinant protein vaccine Razi-COV Pars to enhance mucosal immunity found that it induced mucosal immunity through a recombinant protein strategy, but multiple booster immunizations were required to maintain antibody levels, and no significant increase in sIgA was detected in saliva after population immunization (Fallah Mehrabadi, 2024). In contrast, our mRNA-LNPs vaccine, delivered via just two doses in an IM + IN regimen, elicited high levels of sIgA that persisted for over 4 months without repeated boosting. This notable disparity likely stems from the high *in vivo* antigen expression intrinsic to mRNA vaccines coupled with the adjuvant properties of the LNPs delivery system.

Usually, parenteral vaccines do not induce high levels of potent immune memory at sites of infection, such as tissue-resident memory B cells (BRMs) and T cells (TRMs) or mucosal IgG or sIgA (Mao et al., 2022). Consistent with this, our study shows that IM administration of the PAc mRNA-LNPs vaccine induced a long-term response (≥ 4 months) but a low titer of sIgA in mouse saliva, which may be related to the systemic distribution characteristics of the mRNA vaccine after IM and the fact that only a small amount of the mRNA vaccine is likely to be delivered to the mucosal site where it synthesizes the target antigen protein. Moreover, we found that nasal administration of the PAc mRNA-LNPs vaccine could induce low titers of blood IgA and IgG antibodies but could not induce detectable saliva sIgA and IgG antibodies, which indicates that the mRNA-LNPs vaccine could not activate the nasal-associated lymphoid tissue (NALT) alone, but part of the vaccine enters the blood circulation and participates in systemic immune effects. NALT is a key site of nasal mucosal immunity, capturing vaccine antigens through antigen-presenting cells [such as Membranous cell (M cells), Dendritic cells (DCs) and Intraepithelial lymphocytes (IEL) and activating local B cells to differentiate into sIgA-secreting plasma cells (Madison, 2024)]. So, NALT contains all of the lymphoid cells that are required for the induction and regulation of mucosal immune responses to antigens that are delivered to the nasal cavity (Porgador et al., 1998; Kiyono and Fukuyama, 2004). For example, the intranasal administration of reovirus resulted in the formation of germinal centres in NALT, leading to the clonal expansion of antigen-induced IgA + B cells and the subsequent generation of reovirus-specific sIgA in the respiratory and intestinal tracts (Zuercher et al., 2002). We believe that intranasal administration of the mRNA-LNPs vaccine alone could not maintain the local antigen concentration and thus could not effectively activate the nasal mucosal antigen-presenting cells.

Numerous recent studies have validated the utility of heterologous prime-boost regimens for mucosal mRNA vaccines: IM priming with low-dose mRNA induces robust systemic immunity, which, when combined with mucosal boosting, enhances local immunity, establishes tissue-resident memory cells (TRMs/BRMs), and prevents pathogen colonization—findings supported by work on influenza (Dong et al., 2024) and bacterial mucosal pathogens (Ochsner et al., 2021). Moreover, a recent study showed that systemic immunity can be transformed into local immunity after mucosal enhancement due to immune memory (Ramirez et al., 2024), which may explain the enhanced mucosal immunity observed in the IM + IN group. A promising mucosal immunization strategy for anti-caries vaccines is to induce multiple immune responses by synergistically utilizing mRNA vaccines and multiple vaccine preparations and/or administration routes, thereby improving their protective efficacy and safety. The dose-dependent safety profile observed in this study supports the clinical development of low-dose intranasal boost regimens (e.g., 10 μg or lower) to minimize toxicity, with the selected 10 μg IN boost dose being well-tolerated-showing no detectable local or systemic toxicity beyond transient weight loss at higher doses and thereby strongly supporting its translational potential. Limitations in the safety assessment include the lack of nasal mucosal histological analysis or measurements of systemic inflammatory markers (e.g., cytokines); however, the absence of gross pathological signs or persistent weight loss indicates extremely low local inflammation or systemic toxicity.

Interestingly, our data showed that the IM of the PAc mRNA-LNPs vaccine could induce increased salivary IgG antibodies, and the antibody titer was higher than that of the IM + IN vaccine at some time points. In studies on combined intravascular and nasal administration, IgG antibody titers in exocrine fluids, such as alveolar lavage fluid and nasal lavage fluid, are correlated not only with mucosal immune effects but also with the intensity of systemic immunity induced by intravascular administration (Shi et al., 2012), which may be related to the fact that IgG antibodies secreted from the blood can cross back and forth between the blood circulation and the exocrine fluid via FcRn receptors (Krammer, 2019).

FcRn binds to the IgG Fc region and mediates transepithelial transport, a process essential for IgG homeostasis (Li et al., 2011). Nasal epithelial FcRn exhibits preferential Fc binding at acidic pH, which supports nondegradative vesicular transport across mucosal barriers and subsequent cargo release at physiological pH (Roopenian and Akilesh, 2007); this property improves the stability and half-life of Fc fusion proteins in the nasal mucosa. Furthermore, FcRn is abundantly expressed on antigen-presenting cells (APCs), promoting Fc-mediated antigen delivery (Guilliams et al., 2014). We therefore designed PAc-Fc mRNA-LNP vaccines to enhance mucosal immunity, yet intramuscular (IM) immunization with this construct did not significantly augment mucosal responses, producing only low, sustained sIgA titers similar to PAc mRNA-LNP alone—indicating that systemic Fc-mediated enhancement contributes minimally to mucosal immunity. In contrast, our data directly show that Fc fusion markedly boosted salivary sIgA responses (2.6-fold higher than PAc alone) under the heterologous IM + intranasal (IN) regimen, which correlated with strong inhibition of *S. mutans* biofilm formation and reduced moderate dentinal caries. Based on these findings and published literature, we propose a potential mechanism: during the IN mucosal boost, Fc-FcRn interactions (Roopenian and Akilesh, 2007) may enhance local antigen uptake and presentation by APCs (Guilliams et al., 2014), thereby facilitating B-cell activation and sIgA secretion. However, our study does not provide direct evidence for FcRn-mediated antigen transport or retention, and this mechanistic hypothesis requires further validation using FcRn knockout models or targeted inhibition assays.

To further characterize the immune characteristics of PAc and PAc-Fc mRNA-LNPs vaccines, we examined serum IgG subtypes, including IgG1 and IgG2a, which correspond to Th2- and Th1-skewed immune responses, respectively, and have been shown to play separate and crucial roles in the defence against influenza infection (Collins, 2016). Our mRNA-LNPs vaccine elicited a mixed Th1/Th2 response across different administration routes. However, a Th2 bias (reflected by significantly higher IgG1 than IgG2a levels) was evident, particularly following intranasal-only administration. This bias may be associated with the inflammatory potential of the LNPs components in the nasal mucosa, as reported in preclinical studies (Vadovics et al., 2025). The IgG2a antibody levels in the PAc mRNA-LNPs IM + IN group were greater, and the Th1/Th2 shift was more balanced, which is consistent with the more balanced T-cell response induced by IM + IN immunization reported in the literature (Dong et al., 2024). Moreover, the application of the Fc domain further improved the proportion of IgG2a, and the Th1/Th2 ratio was completely balanced without deviation.

Notably, while caries protection was assessed at a single endpoint (Day 90 post-initial immunization), the sustained high titers of salivary PAc-specific sIgA (>4 months) align with the significant reduction in moderate dentinal caries (>60%), supporting a functional link between vaccine-induced antibody persistence and anti-caries efficacy-consistent with the well-established role of mucosal sIgA in inhibiting *S. mutans* colonization and biofilm formation. We further distinguish antibody persistence from immune memory: antibody persistence refers to the continuous presence of functional antibodies in mucosal secretions (as demonstrated here), providing immediate protective effects, whereas immune memory involves long-lived memory B/T cells that enable rapid recall responses upon re-exposure. Our current conclusions are based on antibody persistence, with immune memory to be validated via antigen re-challenge in future studies.

This study has several limitations: first, immune response analyses focused on antibodies, with no assessments of T-cell subsets, germinal centers, or mucosal memory B cells, so conclusions on immune memory (based solely on antibody durability) should be interpreted cautiously; second, the rat caries model used a small group size (n = 4–5 per group), a pragmatic choice due to ethical constraints and resource limitations, though each study was repeated three times to ensure reproducibility; third, the lack of PAc-only mRNA-LNPs, LNPs-only, and Fc-only/irrelevant mRNA controls limits distinguishing target-specific effects from nonspecific contributions of LNPs or Fc; fourth, direct experimental validation of Fc-FcRn interactions is lacking (e.g., FcRn knockout, binding, or uptake assays), leaving alternative mechanisms (e.g., improved antigen stability) incompletely excluded; fifth, caries protection was evaluated at a single endpoint, and future studies will incorporate multiple time points to more comprehensively delineate the temporal relationship between antibody levels and long-term protective efficacy. Future studies will address these limitations by integrating multi-dimensional immune profiling, expanding animal model group sizes, incorporating complementary control groups, verifying the Fc-FcRn interaction mechanism, and testing the generalizability of IM + IN regimen to other bacterial mucosal pathogens, thereby strengthening the findings’ translational relevance. Beyond the aforementioned limitations, the vaccine’s impact on the oral microbiome and ecological balance remains unaddressed. The oral cavity harbors a symbiotic microbial community where *S. mutans* acts as a cariogen only upon ecological disruption. Though our vaccine-induced sIgA specifically inhibits *S. mutans* biofilm formation, we did not characterize shifts in oral microbial community structure or commensal abundance in immunized animals. This poses a translational ecological risk, as microbiome dysbiosis may impair anti-caries efficacy and predispose hosts to other oral or systemic diseases (Shanmugam et al., 2013). Our follow-up studies will conduct 16S rRNA sequencing, metagenomic analysis, PAc-specific sIgA cross-reactivity validation and vaccine dose/delivery optimization to preserve oral microbial symbiosis.

In summary, the principal advance of this study is the pioneering validation of an mRNA-LNPs platform for mucosal protection against bacterial pathogens—specifically, a PAc-Fc mRNA-LNPs vaccine that induces potent, durable (>4 months) salivary sIgA responses (2.6-fold higher with Fc fusion) via a heterologous IM + IN regimen. The strongest supporting evidence includes robust *in vitro* inhibition of *S. mutans* biofilm formation and >60% reduction in moderate dentinal caries in a preclinical rat model. Prior to clinical translation, concrete next steps will focus on expanding pathogen coverage to clinically prevalent cariogenic bacteria, validating efficacy in humanized FcRn models and non-human primates, evaluating long-term safety in special populations, and assessing impacts on the human oral microbiome. These steps will solidify the translational potential of this mRNA-LNPs platform for dental caries prevention and broader mucosal bacterial infections.

## Data Availability

The original contributions presented in the study are included in the article/Supplementary material, further inquiries can be directed to the corresponding authors.
