# Supplementary material for: Preclinical evaluation of an mRNA-LNPs vaccine for mucosal protection against dental caries
Source: Front Microbiol. 2026 Mar 10;17:1785919. doi: 10.3389/fmicb.2026.1785919 (PMC13008885; doi:10.3389/fmicb.2026.1785919)
Supplement: Supplementary file 1 [file Data_Sheet_1.docx]

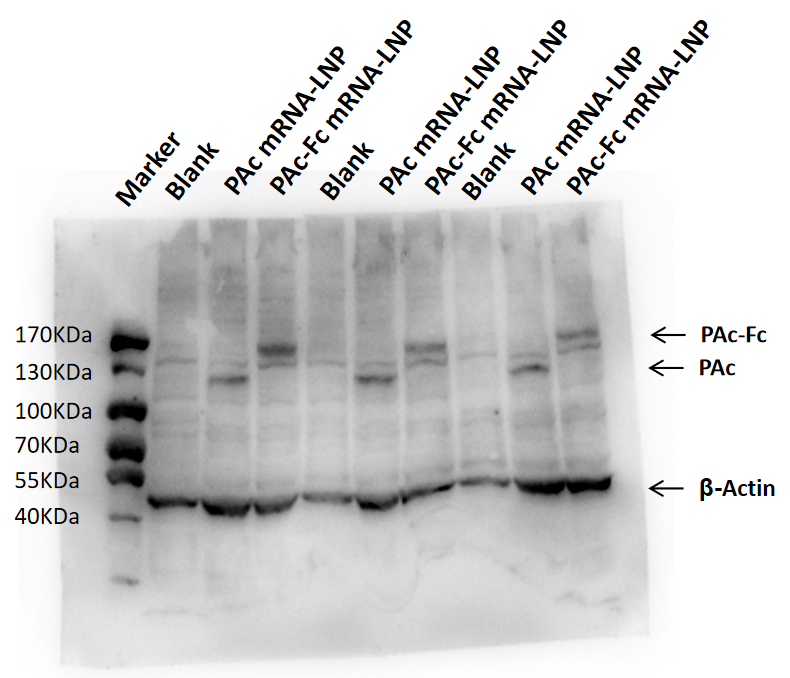


**Supplementary Figure S1**

Western blotting analysis of PAc-mRNA-LNP\PAc-Fc mRNA-LNP expression in 293T cells probed with an anti-HA tag monoclonal antibody. The cells were lysed via RIPA buffer (Cell Signaling Technology) at the indicated time points. The cells were lysed via RIPA buffer (Cell Signaling Technology) at 24 h post-transfection. Twenty micrograms of total cellular lysate were loaded in each lane for SDS-PAGE. The separated proteins were transferred to PVDF membranes for Western blot analysis.


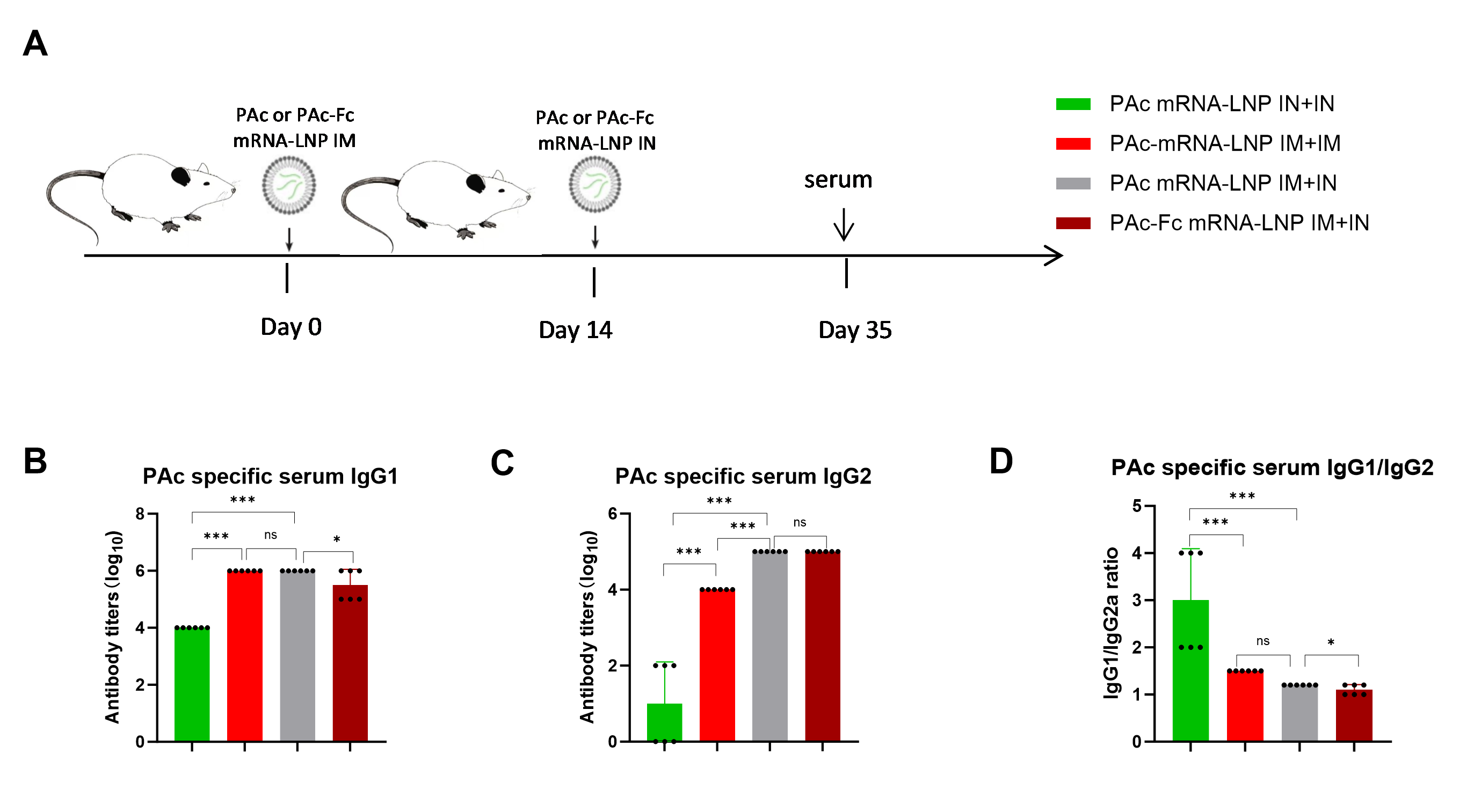


**Supplementary Figure S2**

Influence of IM+IN immunization strategies and Fc-fused antigens on antigen-specific IgG1/IgG2a.

BALB/c mice (n = 6) were vaccinated on an IN (PAc mRNA-LNPs)+IN (PAc mRNA-LNPs)+IM (PAc mRNA-LNPs)+IM (PAc mRNA-LNPs), IM (PAc mRNA-LNPs)+IN (PAc mRNA-LNPs), or IM (PAc-Fc mRNA-LNPs)+IN (PAc-Fc mRNA-LNPs) schedule (days 0, 14) (**A**). Sera were collected 3 weeks post boost. Comparison of the antigen-specific IgG1 (**B**) and IgG2a (**C**) levels and the IgG1/IgG2a ratio (**D**) among the groups. Three independent experiments were carried out in triplicates. Error bars represent standard deviations. All the data were analyzed using the Student's t test; n.s. = not significant, ***P < 0.001.


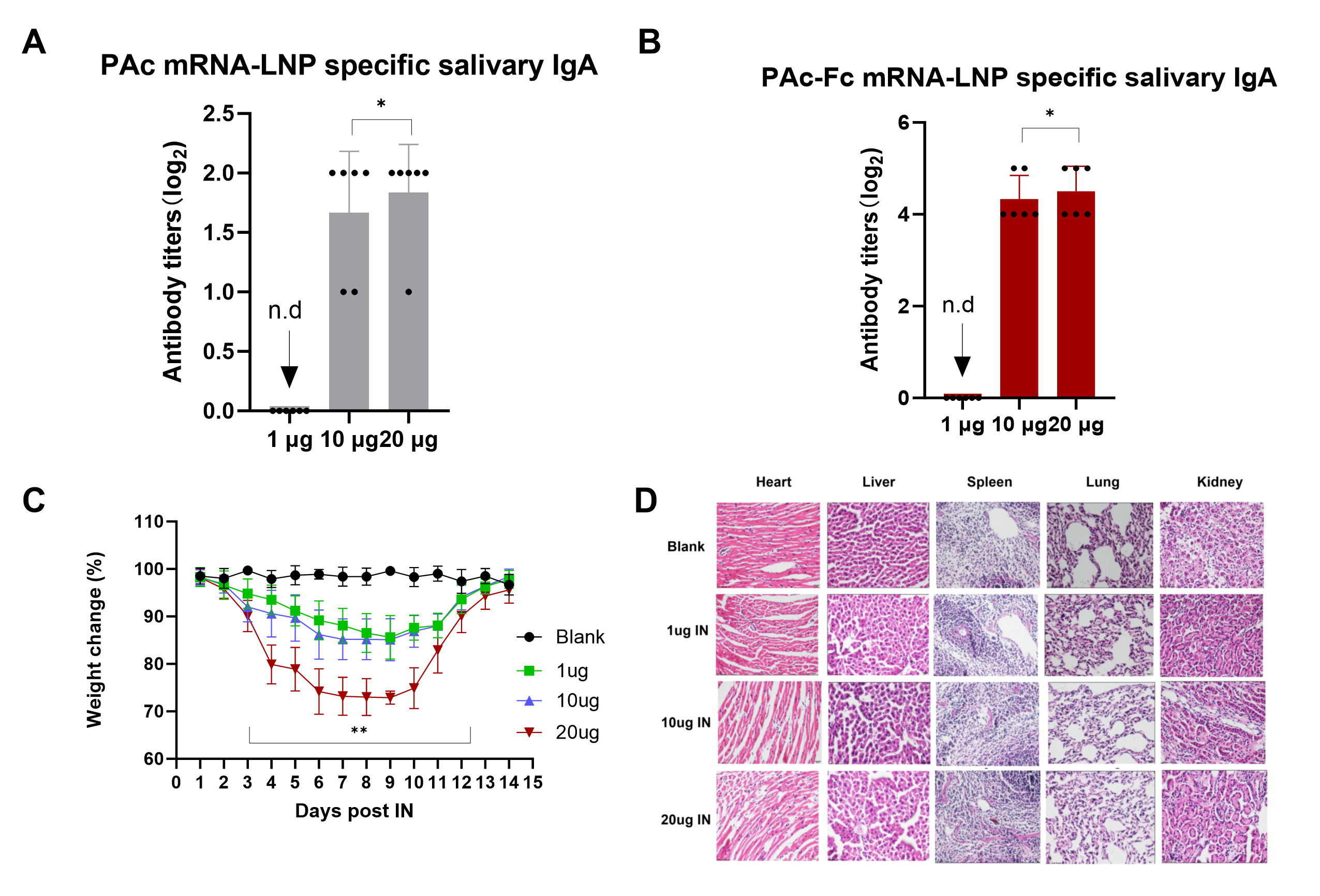


**Supplementary Figure S3**

Measurement of PAc-specific salivary IgA by boosting with 1 µg, 10 µg or 20 µg of PAc mRNA-LNPs IN (**A**) or PAc-Fc mRNA-LNPs IN (**B**) 14 days after 3 µg of IM-primed mRNA-LNPs. Weight change curve of mice after intranasal booster immunization with PAc-Fc mRNA-LNPs (**C**). H&E staining results one week after intranasal booster immunization with PAc-Fc mRNA-LNPs (D). Three independent experiments were carried out in triplicates. Error bars represent standard deviations. All the data were analyzed using the Student's t test; n.d. = not detected, *P < 0.05, **P < 0.01.


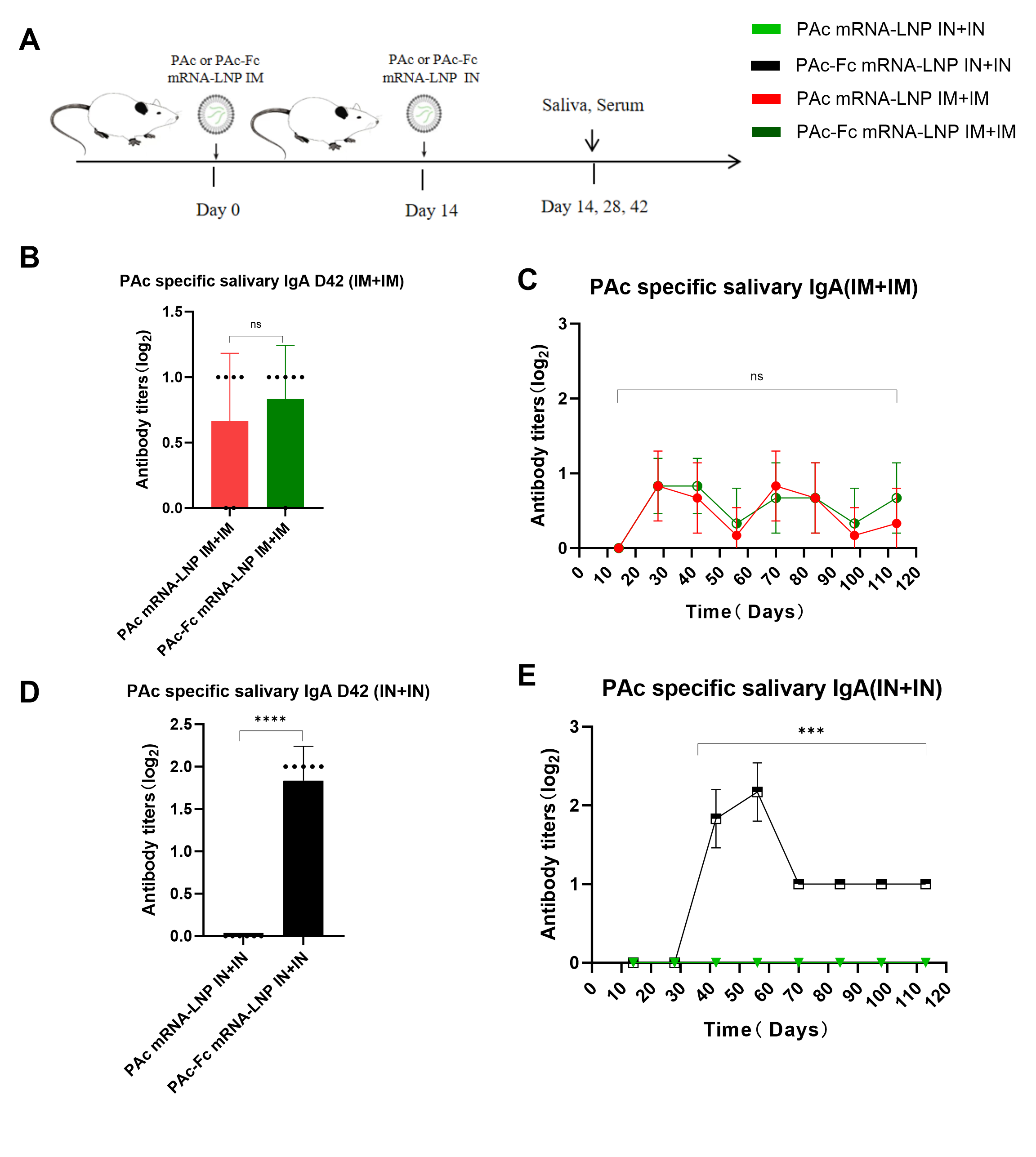


**Supplementary Figure S4**

PAc-Fc mRNA-LNP vaccination enhances PAc-specific sIgA in the IN (prime) + IN (boost) schedule.

BALB/c mice (n = 6) were vaccinated on an IN (prime) + IN (boost) and IM (prime) + IM (boost) schedule (days 0, 14). Mice were IM immunized (**B, C**)with 3 µg of PAc mRNA-LNPs, PAc-Fc mRNA-LNPs or IN immunized (**D, E**)with 10 µg of PAc mRNA-LNPs or PAc-Fc mRNA-LNPs. Saliva samples were collected every 14 days after IM priming for 4 months to assess binding antibody responses. PAc-specific salivary IgA in mice immunized with mRNA-LNPs or PAc-Fc mRNA-LNPs was measured on an IN+ IN or IM + IM schedule. Three independent experiments were carried out in triplicates. Error bars represent standard deviations. All the data were analyzed using the Student's t test; n.d. = not detected, n.s. = not significant, ***P <0.001.

**Supplementary Table S1**

# Table S1 Experimental Groups, Administration Routes and Doses of Anti-caries mRNA-LNP Vaccine

| **Experimental Type** | **Experimental Group** | **Experimental Animal** | **Administration Route** | **Dose** | **Immunization Regimen** |
| --- | --- | --- | --- | --- | --- |
| **Mouse Immunization & Antibody Detection** | PBS Control | 6–8 weeks old female BALB/c mice (n=6/group) | IM+IM; IN+IN; IM+IN | Equal volume of buffer as experimental groups | 2 doses,  2 weeks interval |
|  | PAc mRNA-LNP | 6–8 weeks old female BALB/c mice (n=6/group) | IM+IM | IM: 3 μg mRNA/10 μl Tris buffer | 2 doses,  2 weeks interval |
|  |  |  | IN+IN | IN: 10 μg mRNA/50 μl buffer | 2 doses,  2 weeks interval |
|  |  |  | IM+IN | IM: 3 μg mRNA/10 μl Tris buffer  IN: 10 μg mRNA/50 μl buffer | 2 doses,  2 weeks interval |
|  | PAc-Fc mRNA-LNP | 6–8 weeks old female BALB/c mice (n=6/group) | IM+IM | IM: 3 μg mRNA/10 μl Tris buffer | 2 doses,  2 weeks interval |
|  |  |  | IN+IN | IN: 10 μg mRNA/50 μl buffer | 2 doses,  2 weeks interval |
|  |  |  | IM+IN | IM: 3 μg mRNA/10 μl Tris buffer  IN: 10 μg mRNA/50 μl buffer | 2 doses,  2 weeks interval |
| **Mouse Intranasal Boost Dose Optimization** | PAc mRNA-LNP Low-dose | 6–8 weeks old female BALB/c mice, (n=6/group) | IM (prime) + IN (boost) | IM: 3 μg mRNA/10 μl Tris buffer  IN: 1 μg mRNA/50 μl buffer | 2 doses,  2 weeks interval |
|  | PAc mRNA-LNP Medium-dose | 6–8 weeks old female BALB/c mice, (n=6/group) | IM (prime) + IN (boost) | IM: 3 μg mRNA/10 μl Tris buffer  IN: 10 μg mRNA/50 μl buffer | 2 doses,  2 weeks interval |
|  | PAc mRNA-LNP High-dose | 6–8 weeks old female BALB/c mice, (n=6/group) | IM (prime) + IN (boost) | IM: 3 μg mRNA/10 μl Tris buffer  IN: 20 μg mRNA/50 μl buffer | 2 doses,  2 weeks interval |
|  | PAc-Fc mRNA-LNP Dose Gradient | 6–8 weeks old female BALB/c mice, (n=6/group) | IM (prime) + IN (boost) | IM: 3 μg mRNA/10 μl Tris buffer  IN: 1/10/20 μg mRNA/50 μl buffer | 2 doses,  2 weeks interval |
| **Rat Anti-caries Efficacy Validation** | Blank Control | 18-day-old weaned female SD rats (n=4/group) | - | No immunization | None |
|  | PBS Control | 18-day-old weaned female SD rats (n=4/group) | IM (prime) + IN (boost) | Equal volume of buffer as PAc-Fc group | 2 doses,  2 weeks interval |
|  | PAc-Fc mRNA-LNP | 18-day-old weaned female SD rats (n=4/group) | IM (prime) + IN (boost) | IM: 3 μg mRNA/10 μl Tris buffer  IN: 80 μg mRNA/200 μl buffer | 2 doses,  2 weeks interval |

### Notes

1. IM, intramuscular; IN, intranasal; PAc, Protein antigen c; Fc, fragment crystallizable region of human IgG1; mRNA-LNP, messenger RNA-lipid nanoparticle
2. All mRNA doses refer to the content of pure mRNA; LNP is used as a carrier without separate administration dose.
